# Supplementary material for: Characterizing clinical findings of Sjögren’s Disease patients in community practices using matched electronic dental-health record data
Source: PLoS One. 2023 Jul 31;18(7):e0289335. doi: 10.1371/journal.pone.0289335 (PMC10389720; doi:10.1371/journal.pone.0289335)
Supplement: S1 File — (DOCX) [file pone.0289335.s001.docx]

**Supporting information**

**S1 Table. Questions in nDepth^TM^ to classify Sjögren’s disease (SD) patients based on EHR data.** EHR: electronic health record; mCI: Radioactive iodine dose in millicurie; HIV: Human immunodeficiency virus; IgG4: Immunoglobulin G4-related disease; Anti-Ro/SSA: Antibody to SSA (Ro) antigen; Anti-La/SSB: Antibody to SSB (La) antigen; IU/mL: International units per milliliter; RF: Rheumatoid factor; ANA: Anti-nuclear antibody.

|  | **Categories of questions** | **Survey questions used by reviewers to classify Sjögren’s disease (SD) clinical characteristics** |
| --- | --- | --- |
| 1 | **Specific exclusion conditions** | Does the patient have any of the following conditions?   - - - - History of head and neck radiation treatment (include thyroid cancer patients who underwent radiation/ablation with radioactive iodine(mCi).       - Hepatitis C infection       - Acquired Immunodeficiency Syndrome (HIV)       - Sarcoidosis       - Amyloidosis       - Graft versus host disease       - IgG4-related disease (Example: Mikulicz’s disease)       - Lymphoma       - Primary biliary cirrhosis |
| 2 | **Oral symptoms** | Is information indicating patient's dryness of mouth (dry mouth, xerostomia, taking liquids to swallow food, using sugar free chewing gum, hard candies, sicca symptoms, + dry mouth, sicca syndrome) recorded? |
| 3 | **Ocular symptoms** | Is information on patient’s dryness of eyes  (dry eyes, using eye drops, artificial tears, sicca symptoms, + dry eyes, sicca syndrome) recorded? |
| 4 | **Parotid Gland Enlargement** | Is information on enlargement of parotid glands recorded? |
| 5 | **Antibody to SSA (Ro) antigen**  ≥ 1 IU/mL is positive  < 1 IU/mL is negative  Mention of positive Anti-Ro/SSA | Is a positive serum SSA (Anti-Ro/SSA) antibody recorded? If the lab value isn’t present but the physician states that it is positive, then consider as positive). If the value is present, please enter the value in the comments. |
| 6 | **Antibody to SSB (La) antigen**  ≥ 1 IU/mL is positive  < 1 IU/mL is negative  Mention of positive SSB | Is a positive serum SSB (Anti-La/SSB)( antibody recorded? If the lab value isn’t present but the physician states that it is positive, then consider as positive). If the value is present, please enter the value in the comments. |
| 7 | **Rheumatoid factor (RF)**  > 15; Mention of positive RF | Is a positive rheumatoid factor recorded? (Consider positive if the lab value is greater than 15. If the lab value isn’t present but the physician states that it is positive, then consider as positive). If the value present, please enter this information in the comments. |
| 8 | **Anti-nuclear antibody (ANA) factor**  ≥ 1:160; Mention of positive ANA | Is a positive anti-nuclear antibody (ANA) recorded? (Consider positive if the lab value is greater than or equal to 1:160. If the lab value isn’t present but the physician states that it is positive, then consider as positive). If the ANA value is present, please enter this information in the comment section. |
| 9 | **Minor or labial salivary gland biopsy** | Is a positive labial or minor salivary gland biopsy recorded? (Consider positive if the value of focus score is >= 1 focus / 4mm^2^. If the biopsy report is not present but the physician states that it is positive, then consider as positive). If the value is recorded, please enter this information in the comment section. |
| 10 | **Ocular staining test** | Is a positive ocular staining test recorded? (Consider positive if the value of the ocular staining test score is more than or equal to 3. If the value is not present but the physician states it is positive, consider it positive. If the value is recorded, please enter this information in the comment section. |
| 11 | **Salivary flow test** | Is a positive whole salivary flow test recorded? (Consider positive if the value of unstimulated whole saliva is less than 0.1 ml/min. If the value is not present but the physician states that it is positive, then consider as positive). If the value is recorded, please enter this information in the comment section. |
| 12 | **Supporting information on medications** | Is information on patient taking any following medications present: artificial tears (Brand name: Restasis; Systane), anti-inflammatory eye drops, salivary stimulants or secretagogues (Pilocarpine (Brand name: Salagan), Lifitegrast (XIIDRA), Cevimeline (Brand name: Evoxac), Plaquenil, and/or gustatory stimulation of the salivary glands? |
| 13 | **Classification of SD patients** | **Positive:**  •If there is a presence of dry mouth, or dry eyes, or enlargement of the parotid glands, or supporting information on medications indicating dry mouth or dry eyes.  -AND-  •Presence of at least one objective finding such as positive Anti-Ro/SSA, OR salivary gland biopsy, OR unstimulated salivary test, OR ocular staining test (at least one objective finding with any subjective symptoms).  *Note*: if there are no lab values seen within clinical notes and laboratory records, access Care Web to review patient’s additional information.  **Uncertain:**  •When there is a presence of at least one subjective symptom (dry mouth, dry eyes, enlargement of the salivary gland, or supporting information on medications indicating dry mouth or dry eyes) but has positive rheumatoid factor or positive ANA or positive Anti-La/SSB and no positive Anti-Ro/SSA laboratory value (laboratory tests with negative Anti-Ro/SSA.  **Negative:**  •When there is a mention of “Sjogren’s Syndrome” keyword in a clinician note, however, there is no presence of any subjective or objective findings.  •When there is a presence of one or more subjective findings, there is no laboratory value or other diagnostic test results such as positive minor salivary gland biopsy present to support it.  •No subjective symptoms but only lab findings, even if it included positive Anti-Ro/SSA, or diagnostic test finding.  •Laboratory value RF finding less than 20 (cannot confirm whether it is less than 15 or between 16 to 20); ANA less than 1:160; and/or negative Anti-Ro/SSA and Anti-La/SSB antibodies with presence of subjective symptoms.  •When there is no information present at all/ blank charts. |

**S2 Table. Patients with Sjögren’s Disease (SD), other autoimmune conditions and association based on clinical characteristics’ classification. *** Asterisk indicates statistical significance.

| Autoimmune Diagnosis | Patients with SD diagnosis | | SD groups classified by clinical characteristics | | | | | | |
| --- | --- | --- | --- | --- | --- | --- | --- | --- | --- |
|  | Total | | Positive | | Uncertain | | Negative | | p-value |
|  | N | (%) | N | % | N | % | N | % |  |
| Rheumatoid Arthritis | 94 | (24.9) | 27 | (30) | 27 | (36.5) | 40 | (18.8) | 0.005* |
| Systemic Lupus Erythematosus | 77 | (20.4) | 30 | (33.3) | 21 | (28.4) | 26 | (12.2) | <0.001* |
| Inflammatory Polyarthropathy | 44 | (11.7) | 9 | (10) | 13 | (17.6) | 22 | (10.3) | 0.211 |
| Raynaud's phenomenon | 37 | (9.8) | 11 | (12.2) | 13 | (17.6) | 13 | (6.1) | 0.012* |
| Systemic Sclerosis | 21 | (5.6) | 8 | (8.9) | 6 | (8.1) | 7 | (3.3) | 0.086 |
| Rheumatism, Unspecified and Fibrositis | 19 | (5) | 5 | (5.6) | 2 | (2.7) | 12 | (5.6) | 0.034* |
| Psoriasis | 18 | (4.8) | 6 | (6.7) | 5 | (6.8) | 7 | (3.3) | 0.015* |
| Other Atopic Dermatitis & Related Conditions | 17 | (4.5) | 4 | (4.4) | 3 | (4.1) | 10 | (4.7) | 0.058 |
| Systemic Involvement of Connective Tissue, Unspecified | 17 | (4.5) | 6 | (6.7) | 7 | (9.5) | 4 | (1.9) | 0.001* |
| Chronic Lymphocytic Thyroiditis | 10 | (2.7) | 2 | (2.2) | 4 | (5.4) | 4 | (1.9) | 0.027* |
| Circumscribed Scleroderma | 9 | (2.4) | 1 | (1.1) | 3 | (4.1) | 5 | (2.3) | 0.053 |
| Polymyalgia Rheumatica | 9 | (2.4) | 3 | (3.3) | 1 | (1.4) | 5 | (2.3) | 0.079 |

**S3 Table. Patients with Sjögren’s Disease (SD), comorbidities and association based on clinical characteristics’ classification. *** Asterisk indicates statistical significance.

| Comorbidities | Patients with SD diagnosis | | SD groups classified by clinical characteristics | | | | | | |
| --- | --- | --- | --- | --- | --- | --- | --- | --- | --- |
|  | Total | | Positive | | Uncertain | | Negative | | p-value |
|  | N | (%) | N | (%) | N | (%) | N | (%) |  |
| Pain in joints | 295 | (78.2) | 67 | (74.4) | 60 | (81.1) | 168 | (78.9) | 0.559 |
| Hypertension | 270 | (71.6) | 65 | (72.2) | 48 | (64.9) | 157 | (73.7) | 0.344 |
| Esophageal reflux | 239 | (63.4) | 54 | (60) | 47 | (63.5) | 138 | (64.8) | 0.731 |
| Depressive disorder | 228 | (60.5) | 54 | (60) | 48 | (64.9) | 126 | (59.2) | 0.684 |
| Pain in Limb | 222 | (58.9) | 46 | (51.1) | 42 | (56.8) | 134 | (62.9) | 0.149 |
| Malaise and fatigue | 208 | (55.2) | 42 | (46.7) | 40 | (54.1) | 126 | (59.2) | 0.133 |
| Anemia | 183 | (48.5) | 49 | (54.4) | 30 | (40.5) | 104 | (48.8) | 0.206 |
| Myalgia and myositis/fibromyalgia | 179 | (47.5) | 43 | (47.8) | 43 | (58.1) | 93 | (43.7) | 0.1 |
| Dizziness and giddiness | 168 | (44.6) | 37 | (41.1) | 34 | (45.9) | 97 | (45.5) | 0.751 |
| Hypothyroidism | 149 | (39.5) | 33 | (36.7) | 29 | (39.2) | 87 | (40.8) | 0.792 |
| Hypercholesterolemia | 142 | (37.7) | 23 | (25.6) | 25 | (33.8) | 94 | (44.1) | 0.007* |
| Diabetes | 141 | (37.4) | 29 | (32.2) | 28 | (37.8) | 84 | (39.4) | 0.493 |
| Osteoarthritis | 121 | (32.1) | 38 | (42.2) | 24 | (32.4) | 59 | (27.7) | 0.047* |
| Acute bronchitis | 90 | (23.9) | 18 | (20) | 20 | (27) | 52 | (24.4) | 0.554 |
| Asthma | 66 | (17.5) | 17 | (18.9) | 16 | (21.6) | 33 | (15.5) | 0.453 |
| Pulmonary hypertension | 17 | (4.5) | 5 | (5.6) | 7 | (9.5) | 5 | (2.3) | 0.034* |
| Ocular hypertension | 12 | (3.2) | 0 | (0) | 4 | (5.4) | 8 | (3.8) | 0.069 |
