# Supplementary material for: Characterizing clinical findings of Sjögren’s Disease patients in community practices using matched electronic dental-health record data
Source: PLoS One. 2023 Jul 31;18(7):e0289335. doi: 10.1371/journal.pone.0289335 (PMC10389720; doi:10.1371/journal.pone.0289335)
Supplement: Attachment — Submitted filename: Reviewplosonefeb2023.docx [file pone.0289335.s002.docx]

Review comments

Characterizing Sjogren´s disease patients using electronic health and dental records

The authors end up with concluding that there is a need for establishing criteria to diagnose SD early in community practices. And the study criteria can be used to generate SD cohorts for longitudinal studies.

I can agree on the first statement, but it is a mystery to me how the study criteria developed here can contribute to this?

Introduction

The author intents to develop a paradigm or a screening tool for patients suspect of Mb Sjogren, enforcing the flow of potential patients to specialist care by linking two databases EDR and EHR.

Line 83-89

It is important to notice that Sjogren’s disease is a systemic connective disease not only relying on dryness of mouth and eyes. People have these subjective symptoms for many reasons together with constitutional symptoms without have Sjogren. And although diagnostic criteria are developed, the diagnosis rely on pattern recognition of rheumatologist. The AECG 2002 diagnostic criteria was developed to ensure so homogeneous cohort for research as possible. And internal medical doctors are aware of that Sjogren’s patients are different. Some have very heavy manifestations of the disease including peripheral neuropathy, cutaneous, pulmonary, and renal disease.

In the introduction 4 aims was set-up – have the authors answered them?

Line 113-114

Can the authors comment on the fact that these data bases not are performed as a research database, but for administrative registration and economic properties. Is there a possibility for bias of here?

Line 147 and 250

Eighty-three percent was successfully matched – what about the rest 17%? - Have you any information of the group? Could there be at selection bias here?

Line 164-169

What about smoking status? What about medical treatment? Diuretics? Anti-hypertensive? Anti-depressive? All factors that have a significant impact on the subjective symptoms but also the objective function tests.

Line 175-177 and table 1:

A clear overview of the criteria for categorizing the possible patients complaining of dry mouth or dry eyes into three groups.

Why have you chosen to register the occurrence of Ig M RF? And ANA The occurrence of rheumatoid factor and ANA do not qualify for diagnosis. Noise? See later.

Line 216

Heroic efforts were performed to train the reviewers.

Line 251 and fig 2

441 patients were picked out and 64 patients were excluded resulting in a cohort of 377. 23.9 % positive, 19.6% uncertain and 56.6% negative.

The occurrence of Ig M RF and ANA are the main two laboratory findings that qualify for the patients to be placed into the group of uncertain. Noise?

Line 329

The difference is comorbidity – Is it real or is a consequence of better health care among patients with inflammatory joint or connective disease?

Table 5

The most interesting table. Here we see the results of comparison of the two databases.

Comments of the conformity or lack thereof?

And can you speculate if one of the two databases comes closer to the truth? Or are you comparing to databases with great uncertainties?

Line 360

It is listed that these criteria could be applicable for classifying SS patients using EHR data for research. It is an assertion that cannot stand alone. For me it is not obviously according to the table 5.

S 373-374

This is the hot spot – Serology test can not be used to diagnose Sjogren or to screen for Sjogren.

Line 388-398 – and table 5 and line 403-405

Dry mouth and dry eyes are symptoms that can be treated symptomatically. But there are many reasons for these symptoms at not necessary a result of an ongoing autoimmune process. I am not familiar with your health care system – could there be an economic incitement to give the diagnoses to at patient. Repay for the bill from the dentist or access to be enrolled into clinical randomized trials for new treatments?

Line 403-406 and table 4

The authors are pointing to a very weak point in the daily clinic which are reflected in the records – misdiagnosis or misclassification. Could on of the point in this publication be that there is a urgent need for education.

Line 491-492

It is not clear for me how the criteria developed through this study can be applicable in future research that involves early diagnosis. What are the advantages of these versus the EACG criteria?
